# Investigation of Water Absorption Behavior of Recycled Aggregates and its Effect on Concrete Strength

**DOI:** 10.3390/ma16134505

**Published:** 2023-06-21

**Authors:** Yangfei Ding, Anming She, Wu Yao

**Affiliations:** Key Laboratory of Advanced Civil Engineering Materials of Ministry of Education, School of Materials Science and Engineering, Tongji University, Shanghai 201804, China

**Keywords:** recycled aggregate, water absorption, low-field nuclear magnetic resonance, effective water–cement ratio

## Abstract

The water–cement ratio (w/c) has a significant effect on the strength of recycled concrete. In this study, considering the effects of water/cement ratio, strength, and water content of recycled aggregates, two kinds of pulse sequences of low-field nuclear magnetic resonance (LF-NMR) were applied to investigate the water migration behavior between simulated recycled aggregates (SRA) and water or fresh mortar. Three sets of concrete strength tests were designed and the results were used to verify the findings of LF-NMR imaging tests. The results showed that the depth of water migration in the SRA increases with time: at first the change rate is rapid, then slows down, and eventually tends to remain stable. When the SRA is in contact with fresh mortar with low w/c, no water migration occurs because the hydration of the cement in the mixture consumes a large amount of water, resulting in the inability of water to migrate into the SRA through capillary pressure. For the recycled aggregate concrete with high strength, the addition of extra water will increase the effective w/c and reduce the compressive strength of the concrete.

## 1. Introduction

With the rapid development of the world economy, urbanization and infrastructure construction are carried out on a large scale. Large quantities of construction and demolition waste have been generated in recent years. At present, the disposal of construction solid waste is mainly carried out in a harmless landfill, which is a relatively inefficient way of treatment. Landfilling huge amounts of construction solid waste requires a large area of land and wastes space resources [1]. At the same time, the continuous growth of concrete production also brings huge natural resource depletion problems. With the growing concept of environmental protection, and the increasing policy restrictions on natural stone mining, the actual volume of stone raw materials available for mining will be further reduced. Therefore, the effective use of waste concrete as recycled coarse aggregates (RCAs) can not only solve the problem of recycling construction solid waste, but also reduce the consumption of natural mineral resources, which is of great significance to environmental protection and sustainable development [2].

The RCAs consist of two parts, natural aggregates and adhered mortar, which generally present a higher porosity, higher crushing index, and higher water absorption [3], in comparison with ordinary natural aggregates. Amario [4] studied the mechanical properties of recycled aggregates concrete (RAC) and the results showed that the RCAs have a significant adverse effect on the compressive strength of concrete. Lei’s experimental results show that the compressive strength, flexural strength, and sulfate resistance of recycled concrete are inferior to those of ordinary concrete [5,6,7,8]. The high porosity of RCAs makes them in fresh concrete will absorb the water from the mortar, changing the actual water volume of fresh concrete mixture, so that the slump of fresh concrete is much smaller than that of conventional concrete, and the workability is difficult to reach the design requirement [9]. In addition, the water absorption of RCAs will reduce the w/c in the recycled concrete, which may lead to some of the cementitious materials cannot be fully hydrated [10], as shown by the fast growth of compressive strength within 7 days, but the slow growth of strength after 7 days and the 28-day strength is lower than that of ordinary concrete [11]. Li et al. [12] found that it was likely to achieve the desired compressive strength by adjusting the w/c.

To address the problem of high-water absorption of RCAs, researchers typically add some extra water, so called water absorption compensation (*WAC*) [13], to the mixture proportions of the RAC to compensate for the water loss of matrix caused by RCAs. *WAC* can be applied in two ways: pre-treatment and additional water. Pre-treatment means that the RCAs is soaked to a saturated surface dry (SSD) state before mixing the concrete, or other methods are used to wet the RCAs to a specific moisture content, so that the RCAs lose its ability to absorb water in the mortar. Pre-treated RCAs may not be conducive to the strength of the RAC. As cement hydration proceeds, the internal relative humidity of the RAC gradually decreases, water may migrate from the pre-treated RCAs into the cement slurry, and the w/c of the interfacial transition zone of the RCAs increases, resulting in a decrease in the strength of the interfacial transition zone [14,15]. In addition, the moisture content of the RCAs is difficult to control precisely, so the pre-treatment method is not suitable for practical application. Additional water means that an additional portion of water is added directly to the amount of water used when producing concrete. This method is easy to operate and has little effect on the performance of RAC and is used more widely in research and practical production. The *WAC* for 1 m^3^ RAC is calculated as:

(1) $$WAC=W_{a}\times i$$

where *W_a_* is the aggregate dosage for 1 m^3^ of RAC and *i* is the water absorption rate of RCAs. For concrete in which all aggregates are RCAs, the *WAC* of fine aggregates and coarse aggregates needs to be calculated separately due to the different water absorption rates of fine and coarse aggregates [16].

*WAC* has a significant impact on all properties of RAC. If the *WAC* is not completely absorbed by the RCAs, the excess water will increase the effective w/c in the RAC, which will adversely affect the compressive strength of the recycled concrete [17,18]. The Chinese Standard GB/T 14685-2011 [19] specifies that *WAC* is equal to the water absorption rate of recycled aggregates in 24 h. According to the European standard EN 206 [20], *WAC* is also required for fresh concrete containing coarse lightweight aggregates, and the water absorption rate of lightweight aggregates in fresh concrete is considered to be the value obtained after 1 h of immersion in water. Li et al. [11] innovatively defined the water absorption rate of RCAs from the natural dry state to the SSD state as the effective water absorption rate, and used it as a parameter for the calculation of *WAC*. According to the experimental results, the compressive strength of RAC was reduced by 17–21% when *WAC* was not considered; when *WAC* was considered, the strength of RAC was reduced by 21–43%, but the degree of reduction decreased with the increase of w/c, which may be because the *WAC* calculated based on water absorption does not match the actual water absorption of RCAs in the concrete mixture.

Amario et al. [4] produced concrete with RCAs of different water absorption saturation, and only the mixture with a *WAC* of 50% of the 24 h water absorption shows similar values (strength, workability, and variation of hydration temperature) compared to the natural aggregate concrete: it indicates that the effective w/c of these mixtures is similar. Therefore, 50% water content is used as a parameter for the *WAC* calculation, when extra water does not affect the water available in the fresh concrete. Leite et al. [21] stated that the amount of *WAC* calculated at 80% and 90% of 24 h water absorption of RAC would be feasible for both compressive strength and workability. Mefteh et al. [22] prepared RAC with extra water based on 80% of its saturated water absorption. Agrela [23] recommended 92% of its 24 h water absorption was adopted as extra water in the concrete mixture. These reports suggest that there is no consensus on the appropriate value for the *WAC*.

In practice, these *WAC* values are not necessarily appropriate for concrete with different w/c, and these experiments do not demonstrate whether the actual w/c in fresh concrete mixtures is the design value [24]. In fresh recycled concrete, the water absorption of RAC and the hydration behavior of cement occur simultaneously, and the hydration of cement consumes water, resulting in less water absorbed by RCAs, so the *WAC* calculated by water absorption does not necessarily match the actual situation, and few researchers have conducted systematic quantitative studies on the actual water absorption behavior of RCAs. The biggest difficulty to investigate the water absorption behavior of RCAs in fresh concrete is the long duration of cement hydration and the difficulty to characterize the distribution of water in recycled aggregates after cement setting [25]. Zhen Li [26] sieved the cement paste from the fresh mortar before initial setting and calculated the water absorption rate of the recycled fine aggregate in the paste by drying and comparing the difference in the total water content of the paste between the mixture containing the recycled fine aggregate and the reference cement paste. This method is only applicable to the cement paste before initial setting and does not allow for continuous monitoring of the water absorption behavior. However, this method can only monitor the change of w/c of cement paste before final setting and cannot achieve continuous monitoring for a longer period.

LF-NMR is a new method to measure the hydration process of cement and to analyze the moisture distribution in cementitious materials [27]. The process of nuclear spins recovering from the non-equilibrium state at high energy level to the equilibrium state at low energy level after the effect of RF field is called relaxation. LF-NMR measurement targets hydrogen protons in water molecules, whose relaxation behavior releases pulses that can be monitored by LF-NMR. The relaxation signal intensity and relaxation time are the main indicators. The amount of its relaxation signal can reflect the amount of water molecules, and the different relaxation times can reflect the different states of water, and then characterize the information of internal microstructure and water distribution of materials, so it can qualitatively and quantitatively study the behavior of water absorption in cementitious materials. The advantages of LF-NMR as a non-invasive test method that allows continuous measurements have led to its wide application in cement hydration and water absorption behavior studies. She et al. [28,29] used LF-NMR to monitor the effect of nano-SiO_2_ and w/c on the early hydration process of cement. Gummerson et al. [30] applied LF-NMR for the first time to measure the internal water distribution of porous cementitious materials. Kopinga and Pel [31] used LF-NMR to monitor the distribution of moisture in a single direction with time during drying and absorption of brick samples, respectively. Leech et al. [32] observed the water distribution of concrete in a single direction water migration process by using LF-NMR. Zhang et al. [27,28,29] analyzed the migration process of water in the pores of artificial sandstone samples using T_2_ spectra by LF-NMR.

Too little research has been focused on the water absorption between RCAs and fresh mortar. Measuring the water absorption behavior of RCAs in fresh concrete by LF-NMR imaging can provide a basis for accurate *WAC* calculation. In this study, considering the effects of w/c, strength, and water content of recycled aggregates, two kinds of pulse sequences of LF-NMR imaging were applied to investigate the water migration behavior between SRA and water or fresh mortar.

## 2. Materials and Methods

### 2.1. Materials

The white Portland cement used for the samples in LF NMR imaging was P.W. 42.5 cement and the cement used for fresh mortar was P.W. 52.5 cement. The chemical composition of these cements is shown in Table 1. The fine aggregates used in the test samples were standard sands with particle sizes ranging from 0.07 to 2.00 mm. In order to improve the flowability of fresh mortar in the sample bottles, polycarboxylate superplasticizer (PS) was also used in the tests.

The cement used in the concrete experiments was P.Ⅱ 52.5 cement. 95U grade silica fume was used as the mineral admixture. The RCAs used in the experiment was obtained from a factory of Shanghai Urban Construction Materials Co. Ltd. (Shanghai, China), which were divided into RCA-1 and RCA-2 according to the particle size interval. The physical properties of RCAs are shown in Table 2. The coarse aggregate contains 55% RCA-1 and 45% RCA-2. River sands were used as fine aggregates with a fineness modulus of 2.63.

### 2.2. Mix Proportion and Sample Preparation

#### 2.2.1. Specimen Preparation for 1D Frequency Coding Sequence Test

As ordinary Portland cement contains ferromagnetic material, which disturbs the signals collected by the LF-NMR analysis and imaging system, so the recycled aggregates obtained from waste concrete cannot be measured by the LF-NMR instrument. In addition, the irregular shape of the recycled aggregate makes it difficult to accurately measure the distribution of water in the interfacial transition zones of the RCAs, so a kind of simulated recycled aggregate (SRA) prepared from white cement was used as test samples to simplify the water migration of the recycled aggregates in the one direction. The attached mortar in the surface of the real recycled aggregate plays a decisive role in water absorption, so the water absorption of natural aggregate can be disregarded.

The molds used for sample preparation were colorless transparent bottles made of PET with a height of 85 mm and an aperture of 55 mm. The interior of the molds was polished to avoid gaps between mortar and molds.

Two types of SRA were used for the experiments, C25 and C40. C25 and C40 were designed to simulate the mortar in concrete with compressive strengths of 25 MPa and 40 MPa, respectively. The w/c of C25 was 0.55 and the sand–cement ratio was 1.3. The w/c of C40 was 0.4 and the sand–cement ratio was 1.3.

**Figure 1:**
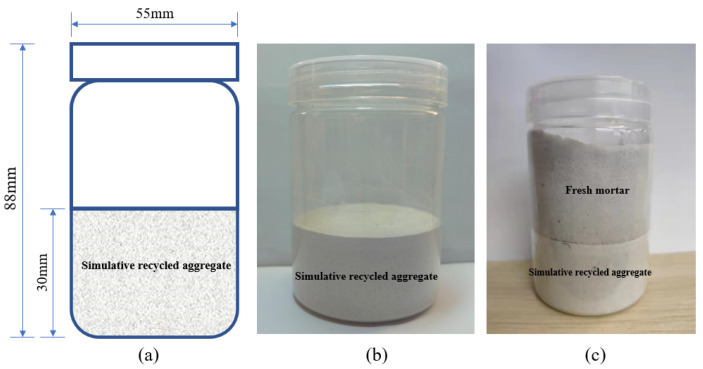
LF-NMR imaging samples: (**a**) schematic; (**b**) sample after molding; (**c**) sample after adding fresh mortar.

The cement, sand, and water were weighed. After mixing cement, sand, and water for 120 s, the mixture was cast into molds and vibrated for 10 s to make the mixture level. The height of the samples was 30 mm. The samples were cured in a fog room at constant temperature and humidity, where the temperature was 20 ± 2 °C and the relative humidity was ≥95%. One day after curing, the samples were placed in saturated calcium hydroxide solution for 180 days to ensure that cement were close to full hydration. Finally, the samples were dried in a drying chamber at 30 °C until the mass no longer changed, and then polished to remove the mortar adhering to the mold. The samples are shown in Figure 1.

#### 2.2.2. Specimen Preparation for CPMG Pulse Sequence Test

If the recycled aggregate absorbs water in the cement paste, then the effective w/c of the cement paste is changed and the hydration process is subsequently affected. In order to investigate the factors influencing the water absorption behavior of SRA, three series of specimens were prepared separately, considering the w/c of cement paste (0.3, 0.5), water content of SRA (dry, SSD), and the type of SRA (C25, C40). The sample compositions are shown in Table 3. For example, WC03-D-C25 indicates dry C25 submerged in a cement paste with a w/c of 0.3.

The SRA was crushed in advance into particles with a particle size of 5 mm. A portion of the dried SRA is submerged in water for 24 h, then the SRA is removed from the water and the surface of the SRA is sufficiently dried with a towel until the water film on the surface of the SRA disappears in order to obtain the SRA in a saturated surface dry state.

#### 2.2.3. Mix Proportions of Concrete

To verify the accuracy of the LF-NMR measurement of the water absorption behavior of recycled aggregates, a set of experiments was designed to compare the effect of different *WAC* on the compressive strength of concrete. The concrete experiments consisted of three groups: no *WAC* added (RAC-1), *WAC* combined with water added together during concrete mixing (RAC-2), and recycled aggregates wetted in advance by *WAC* in the form of spray (RAC-3). For RAC-3, the weighed *WAC* was sprayed on the surface of the recycled aggregate, turned to wet the recycled aggregate evenly, and then sealed for 30 min to make the recycled aggregate absorbing water [33]. The *WAC* is calculated as shown in Equation (1). Water absorption measurements consisted of complete immersion of the samples in water for 24 h. The mass of each sample was greater than 4 kg to avoid as much as possible the difference in water absorption between each recycled aggregate. After 24 h of water immersion, the samples were treated to a saturated face-dry state. Finally, the samples are dried at a specific drying temperature to remove all moisture from the pores (*M_dry_*). The results of the study by Théréné et al. [34] indicated that drying above 30℃ caused dehydration of Ettringite, which increased the results of the water absorption test, so in this study the drying temperature was selected to be 30 °C and the samples were dried for 40 days until a constant weight was obtained. The formula for calculating water absorption is as follows:

(2) $$WA=\frac{M_{SSD}-M_{dry}}{M_{dry}}\times 100\%$$

The mix proportions of the three groups of concrete are shown in Table 4. The basic w/b is 0.24. After adding *WAC*, the actual w/b of RAC-2 and RAC-3 is 0.32. Moreover, the slump of concrete was designed to 180 mm.

The concrete mixture was prepared by the following procedure: Firstly, all powder materials, water, and PS were added, mixing for 5 min. Later, the RCAs were added, mixing until homogeneous. Finally, the river sand was added, mixing it for 5 min, after which the mixture was prepared.

### 2.3. Methods

#### 2.3.1. LF-NMR Test of 1D Frequency Coding Sequence

**Figure 2:**
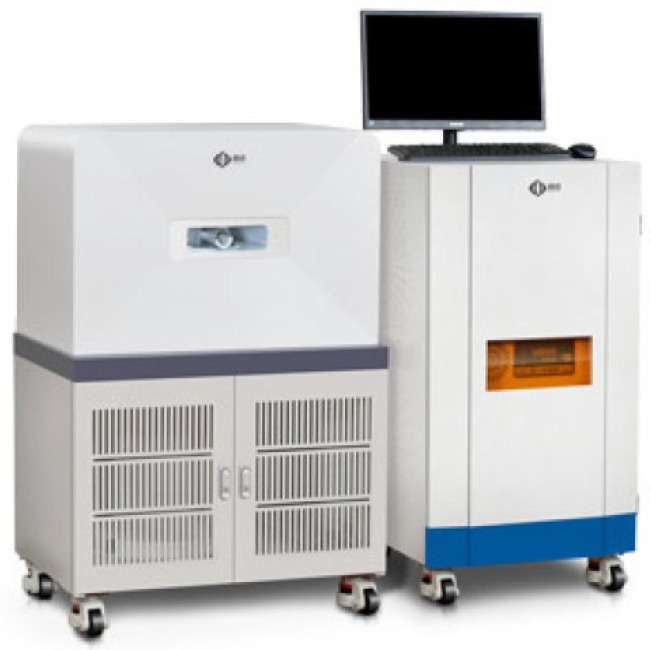
Medium-sized NMR analysis and imaging system.

The LF-NMR instrument was provided by Suzhou Niumag Analytical Instrument Corporation (Suzhou, China), model MesoMR23-060H. As shown in Figure 2, the magnet system contains a permanent magnet with a magnetic field strength of 0.5 T. The main frequency of the instrument is 21.3 MHz.

**Figure 3:**
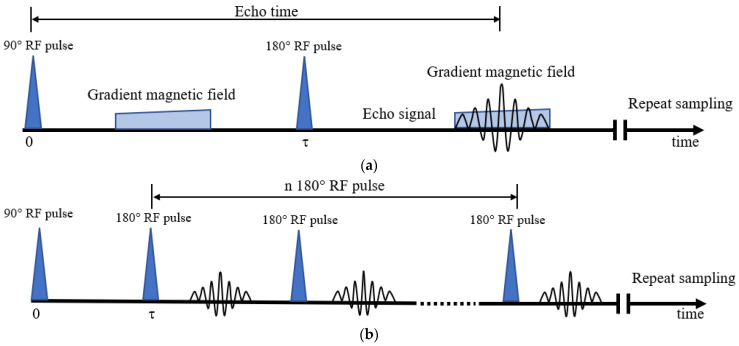
Sequences of LF-NMR measurement used in this study: (**a**) 1D frequency coding sequence; (**b**) CPMG sequence.

To obtain information on the moisture content of the cross-section at different locations in the sample, 1D frequency coding sequence of LF-NMR was applied. As shown in Figure 3a, the 1D frequency coding sequence is a series of RF pulses: first a 90° RF pulse is applied, after a certain time interval (τ) a linear magnetic field gradient pulse is applied, then a 180° RF pulse is applied. Finally, another linear magnetic field gradient pulse is applied, and the signal is acquired at this point [35]. Because the magnetic field gradient is linear, each specific magnetic field intensity corresponds to a specific location in the sample, and the moisture content is measured according to the maximum signal amplitude of the different locations. By applying the Fourier transform, the signal intensity can be calculated according to the following equation:

(3) $$A=A_{0}\left[1-\exp\left(\frac{-T_{R}}{T_{1}}\right)\right]exp\left(\frac{-T_{E}}{T_{2}}\right)$$

where *A* is the intensity of the signal at an individual point, *A*_0_ is the proton density at an individual point, *T_R_* is the repeat time, *T_E_* is the echo time, *T*_1_ is the longitudinal relaxation time, and *T*_2_ is the transverse relaxation time [36].

The 1D frequency coding sequence simultaneously acquires information on the moisture content of 512 different cross-sections within the sample, which is used to analyze the moisture migration behavior of samples.

The composition of fresh mortar is P.W 52.5 white cement, river sand, and water, where the w/c is 0.3 and 0.5, sand–cement ratio is 1.2, and PS content is 0.01%. The cement, sand, and water were weighed. After mixing cement, sand, and water for 60 s, the mixture was cast into samples, controlling the height of the fresh mortar to 30 mm (shown in Figure 1c). The sealed samples were placed in the NMR analysis and imaging system, where the parameters were set as follows: sampling frequency of 50 kHz, accumulation number of 64, echo time of 2.50000 ms, sampling interval of 10 min, and continuous sampling for 24 h. Moreover, 1D frequency coding sequences were tested on three qualified samples for replication.

The relationship between the signal intensity of water at position x and position x were plotted for different moments, thus reflecting the pattern of water migration of the sample after contact with fresh mortar. As a reference, the above experiments were repeated after replacing the fresh mortar with water to simulate the moisture migration behavior occurring between the recycled aggregate and water.

#### 2.3.2. LF-NMR Test of CPMG

The CPMG pulse sequence was used to measure the transverse relaxation times (T_2_) and ^1^H signal amplitude for hydrating sample. As shown in Figure 3b, by applying multiple 180° RF pulses, signal attenuation due to uneven magnetic fields was retarded and interference was eliminated. Since cement-based materials generally consist of pores of different sizes, the total relaxation *M*(*t*) measured by the CPMG sequence is a superposition of the relaxations in different pores:

(4) $$M\left(t\right)=\sum_{i}A_{i}\exp\left(\frac{-t}{t_{2i}}\right)$$

Mix the weighed cement with water and stir manually for about 1 min, then immediately pour it into the SRA and shake it slightly to submerge the SRA into the paste. The echo interval time of the LF-NMR instrument was set to 0.18 ms, and the rest of the parameters were set as for the 1D frequency coding sequence. CPMGs were tested on three qualified samples for replication.

The analysis of T_2_ allows monitoring the change of the effective w/c in the sample and thus analyzing the water absorption behavior of SRA in the cement paste.

#### 2.3.3. Slump and Compressive Strength Test

The slump test is used to evaluate the workability of concrete mixture, of which the steps conform to the Chinese standard GB/T 50080-2016 [37]. The mixture for each group was cast in three steel molds of 100 × 100 × 100 mm and then compacted on a vibrating table. One day after casting, the concrete samples were demolded and submerged in water at 20 ± 2 °C for 7 and 28 days to cure. The compressive strength of the recycled concrete samples was measured according to the Chinese Standard GB/T 50081-2019 [38].

## 3. Results and Discussion

### 3.1. Experimental Results from 1D Frequency Coding Sequence

**Figure 4:**
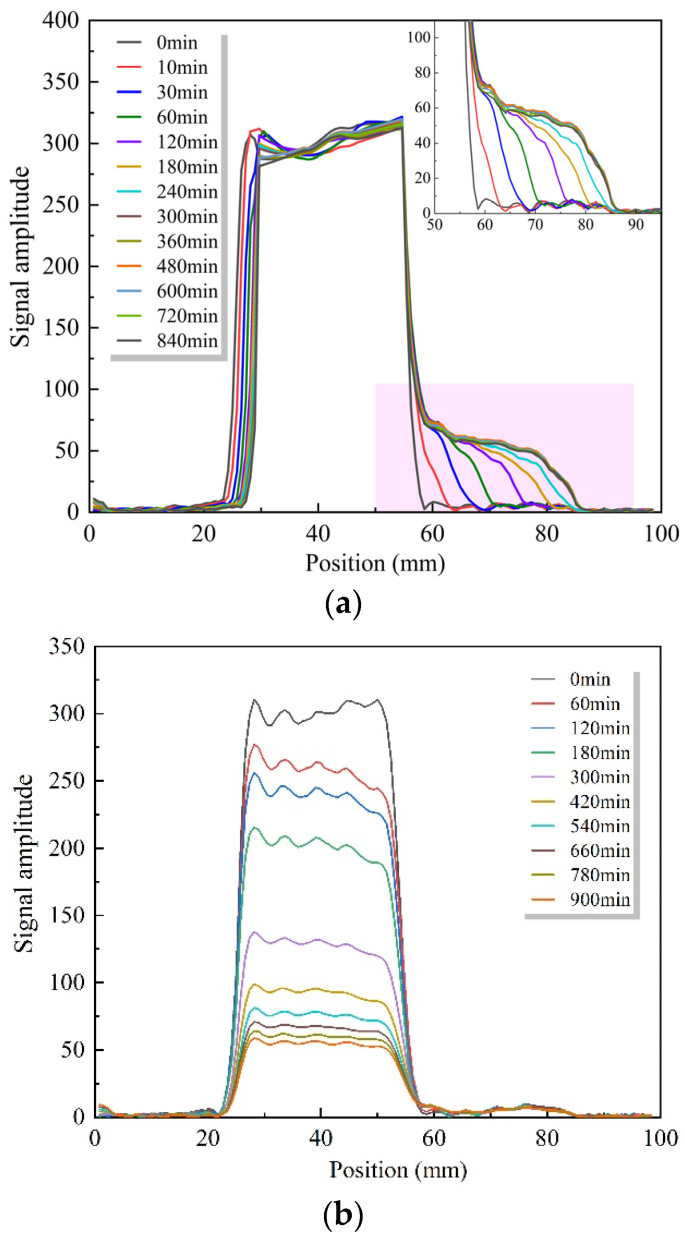

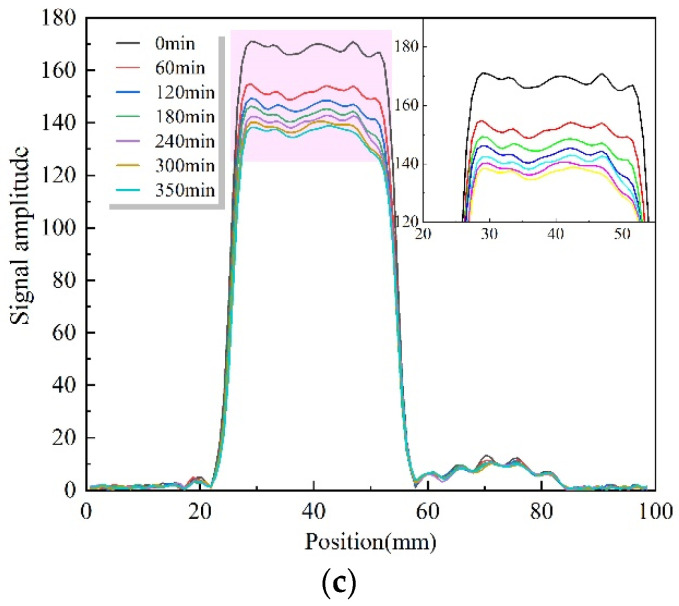
Moisture distribution of different specimens: (**a**) C25 in water; (**b**) C25 in WC05; (**c**) C25 in WC03.

The relationship between the intensity and location of the moisture signal within the sample after different water absorption times is shown in Figure 4. The axes on the figure correspond to fresh mortar or water at 25–55 mm and SRAs at 55–85 mm. The signal intensity inside all samples was not equal to zero at the beginning of the measurement, indicating the presence of small amounts of evaporable water in the samples even after drying.

As shown in Figure 4a, the moisture migration occurs immediately after the contact between C25 and water, and moisture has penetrated about 5 mm at 10 min. Moisture penetration within the recycled aggregate gradually deepens with time and the curve continues to shift to the right while the slope gradually decreases. The overlap of the moisture distribution curves appeared after 300 min, indicating that the water absorption process had ended and the moisture distribution inside the SRA reached equilibrium. The curve in the 20–30 mm interval on the coordinate axis corresponds to the liquid level of the water above the sample. With the water migrating at the interface between the recycled aggregate and the water column, the upper layer of water gradually decreases and the liquid level decreases.

The moisture distribution curve of RAC absorbing water from the mortar with w/c = 0.5 is shown in Figure 4b. As the cement hydrates, the free water in the fresh mortar is gradually converted into bound water in the hydration products, which leads to a weakening of the moisture signal intensity in the fresh mortar, resulting in a gradual decrease in this section of the curve. Meanwhile, it can be observed that there is no change in the curve inside the SRA from 0 to 900 min, indicating that no migration of water to the inside of the recycled aggregate is monitored.

The moisture distribution curve of RAC absorbing water from the mortar with w/c = 0.3 is shown in Figure 4c, and again no sample was monitored to absorb moisture from the fresh mortar.

**Figure 5:**
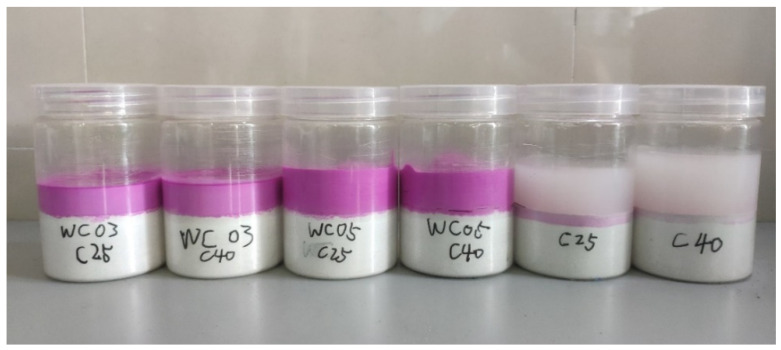
Experimental phenomenon of SRA macroscopic water absorption.

Phenolphthalein was added to the upper layer of fresh mortar or water as a color indicator. The experimental phenomena of the sample after water absorption for 6 h is shown in Figure 5. At this time, water penetration was about 6 mm in C25 and 4 mm in C40, while almost no water penetration was observed in all four samples added to the fresh mortar. The experimental phenomena coincide with the LF-NMR test results. Table 5 shows the result statistics of whether water migration occurs in mortar or water for SRA.

In summary, the water migration behavior of SRA in fresh mortar is very different from the water absorption behavior of SRA with pure water.

There are several possible reasons for the test results of 1D frequency coding:

(1) In order to obtain water signals in pores with pore size ≤ 100 nm, the echo interval time of the LF-NMR instrument should be set below 60 μs [29]. However, due to the limitation of the 1D frequency encoding sequence itself, the echo interval time in this experiment was set to 2.5 ms, so there is a possibility that a part of the signal from tiny pores was lost. There may be very little water migration between the SRA and fresh mortar, and the water preferentially enters the small pores inside the SRA and thus cannot be monitored. In contrast, there is a significant amount of water migration between the SRA and water, which is not affected by the accuracy of the measurement.
(2) In high-strength recycled concrete with compressive strength greater than 60 MPa, the commonly used cement grades are P Ⅰ 52.5 or P Ⅱ 52.5, in which the clinker content is greater than 90% and the particle size is around 20 μm, resulting in fast cement hydration. After the contact between recycled aggregate and fresh mortar, the process of water absorption by the recycled aggregate and the process of cement hydration are carried out simultaneously, and the cement hydration consumes a large amount of free water rapidly. The w/c of high-strength recycled concrete is between 0.2 and 0.3, which is close to the theoretical minimum w/c required for full hydration of cement [39]. The rapid hydration of the cement leads to a reduction in the amount of water available in the mortar for absorption by the recycled aggregate, resulting in negligible actual water absorption by the recycled aggregate [33].
(3) There are three main modes of water migration in silicate materials, including capillary processes, diffusion processes, and permeation under pressure gradients [40]. For unsaturated silicate materials, the capillary process is the most dominant migration mode. The relative humidity of water can be considered to remain constant when the recycled aggregates are in contact with pure water. Moisture migrates into the recycled aggregate under diffusion and capillary pressure until it fills all the open pores inside the recycled aggregate, which is the saturated state of water absorption of the recycled aggregate [41,42]. For the process of contact between the recycled aggregate and fresh mortar, the relative humidity of the fresh mortar decreases with its own hydration, and there may be a trace of water absorption of the recycled aggregate to make the internal humidity rise. At a certain moment, the relative humidity inside and outside the recycled aggregate reaches equilibrium, which is the maximum limit of water absorption of recycled aggregate in the mortar, and this maximum limit is much smaller than the saturated state of water absorption of recycled aggregate [43].
(4) In high-strength concrete, the mortar has low porosity and few connecting pores, resulting in less migration of moisture within the mortar. After the recycled aggregate absorbs a trace amount of water from the surrounding mortar, the surrounding fresh mortar needs to be replenished with water from the more peripheral mortar by diffusion [44], which makes the amount of water available in the mortar for absorption by the recycled aggregate within a certain period of time much smaller than the amount of water that can be absorbed by the recycled aggregate in water [45].

In summary, although recycled aggregates are characterized by high water absorption, recycled aggregates in fresh concrete are possibly unable to absorb water from fresh mortar or have extremely weak water absorption behavior.

### 3.2. Experimental Results from CPMG

According to the testing principle of LF-NMR, there is a positive relationship between the obtained signal amplitude and the evaporable moisture content of the SRA. However, the initial signal amplitude was different for each sample due to the different amount of cement paste. In order to exclude the effect of the difference in cement paste dosage on the signal amplitude, the signal amplitude obtained for each sample was divided by the initial signal amplitude and then converted to a percentage, thus replacing the signal amplitude with the signal intensity [46].

**Figure 6:**
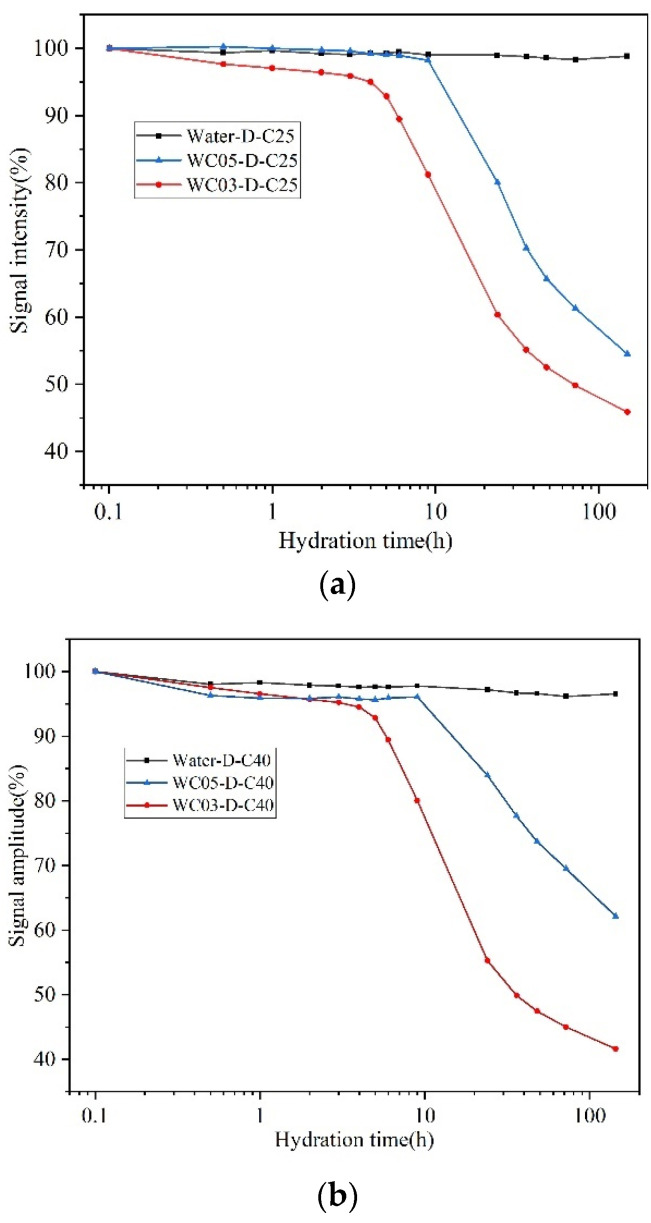
Hydration curves of SRA in water and cement paste of different w/c: (**a**) C25; (**b**) C40.

Figure 6a shows the signal intensity results of C25 during hydration in water and cement paste up to 150 h. The signal intensity remains smooth when water and RCA are in contact, which is because water only migrates into the SRA and no chemical reaction occurs, thus the signal intensity of water does not change. The stable signal intensity of water also indicates that water migration does not cause signal loss, which proves the accuracy of the CPMG sequence test. There is a similar trend in the signal intensity for all cement paste samples. As cement hydration proceeds, a portion of water gradually enters the pores of the hydration products, and this portion of water can be monitored by the CPMG sequence [47]. The remaining portion of water is converted to chemically bound water after participating in the hydration reaction, which causes the signal intensity of moisture to decrease gradually with time.

The signal intensities of WC03-D-C25 and WC05-D-C25 were very close to each other and remained stable within 5 h at the beginning of the test, because both were in the induction period of hydration. After 5 h, the curve of WC03-D-C25 was the first to show an inflection point and the slope of signal decay was greater, which indicated that the hydration rate of WC03-D-C25 was greater than that of WC05-D-C25. According to ref. [46,48], high w/c dilutes the OH^−^ and Ca^2+^ ions in the cement paste, thus delaying the reaction rate of hydration. At the end of monitoring, the signal intensity of WC05-D-C25 still maintains a decreasing trend, while the decreasing trend of the curve of WC03-D-C25 has diminished. Therefore, it can be inferred that the cement paste with w/c = 0.5 may be more fully hydrated in the subsequent hydration stage because of the presence of sufficient water compared to the paste with w/c = 0.3.

Figure 6b shows the signal intensity results of C40 during hydration in water and cement paste up to 150 h, which is very similar to the pattern exhibited by C25.

**Figure 7:**
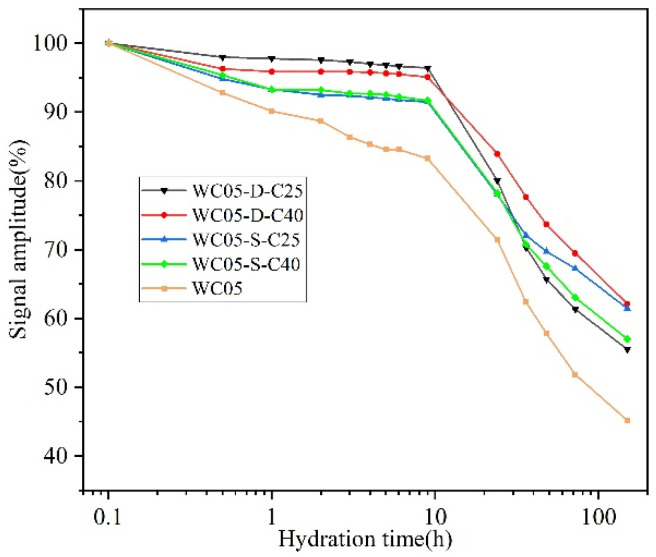
Effect of different SRA on the hydration of cement paste with w/c = 0.5.

Figure 7 shows the signal intensity results after the addition of SRA with different water contents and strengths to a cement paste with w/c = 0.5. WC05 is the pure cement paste hydration curve. If SRA does not absorb water from the paste and the w/c of the paste remains constant, then all other curves should overlap with WC05; however, the test results are clearly not the case. The addition of SRA slows down the signal intensity attenuation to varying degrees compared to WC05.

Compared to WC05, the cement paste hydration curve with the addition of dry SRA has a greater slope, which means that the cement paste hydrates faster. The dry SRA absorbs water in the paste with a large w/c, which decreases the effective w/c and accelerates the hydration of cement. In contrast, the slope of the hydration curve of the cement pastes with the addition of saturated SRA decreased, probably because a small amount of water remained on the surface of the saturated SRA, resulting in a locally larger w/c. At the end of the monitoring, the signal intensity of the sample with saturated SRA was higher than that of the sample with dry SRA, because more water was retained within the saturated SRA.

Compared to C40, C25 has a higher water absorption rate and absorbs more moisture in the paste, resulting in a more pronounced acceleration of hydration, so the slope of WC05-D-C25 is greater than that of WC05-D-C40. Comparing WC05-S-C25 and WC05-S-C40, it can be seen that the difference between the two curves is small, which indicates that the strength of the aggregate in the saturated case on the moisture migration can be disregarded.

**Figure 8:**
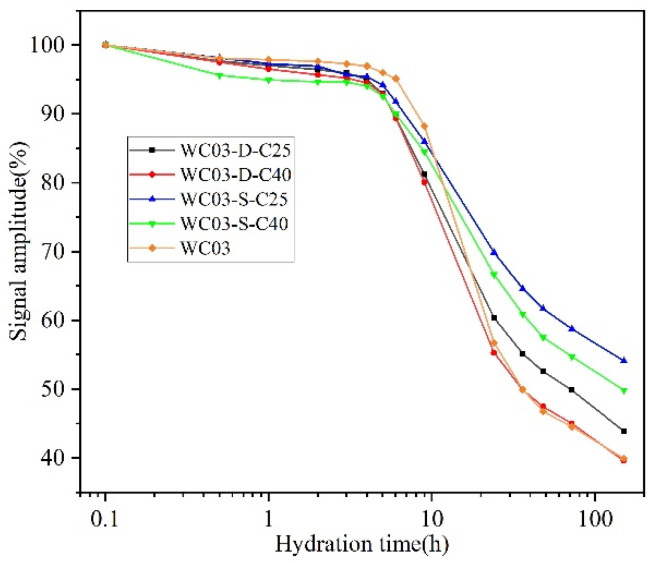
Effect of different SRA on the hydration of cement paste with w/c = 0.3.

Figure 8 shows the signal intensity results after the addition of SRA with different water contents and strengths to the paste with w/c = 0.3. Apparently, the effect of SRA with different strengths and water contents on the early hydration of the cement paste with w/c = 0.3 in 9 h is less than in the cement paste with w/c = 0.5, which is due to the low w/c in the paste with w/c = 0.3, where the cement hydrates quickly, consumes most of the water, and can migrate less water. Apart from this, the effect of SRA on the signal intensity of WC03 reflects the same pattern as in WC05. It is particularly noteworthy that at the end of the measurement, WC03-D-C40 and WC03 have the same signal intensity, which indicates that both cement pastes have reached the same hydration level and C40 does not absorb moisture from the paste. In short, dry C40 does not change the w/c of the paste.

### 3.3. Workability

The measurement results of LF-NMR imaging showed that the recycled aggregate absorbed negligible water in the high-strength recycled concrete, so that the *WAC* added during the mixing of the recycled concrete was in fact not absorbed by the recycled aggregate, which led to an increase in the actual w/c of the recycled concrete and had a detrimental effect on the compressive strength of the concrete [49]. Three sets of concrete with different water consumption and mixing methods were designed to verify this idea.

**Figure 9:**
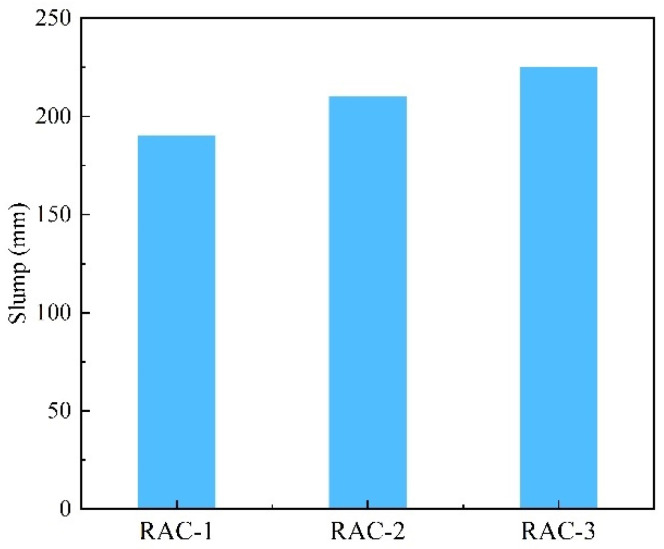
Slump of fresh concrete.

The slump results of the RAC are shown in Figure 9. The slump of all three groups of mixtures could reach the design target after the addition of PS. Although RAC-1 had the highest amount of PS, the slump was significantly lower than RAC-2 and RAC-3, which was due to the lower water consumption of RAC-1, while the w/c was the main factor to determine the slump of concrete. After adding the same amount of *WAC*, the slump of both RAC-2 and RAC-3 was significantly improved, and the improvement of RAC-3 was greater than that of RAC-2, which was due to the existence of the water film on the surface of recycled aggregate when wetting it in advance, which played the role of lubrication [50].

### 3.4. Compressive Strength

**Figure 10:**
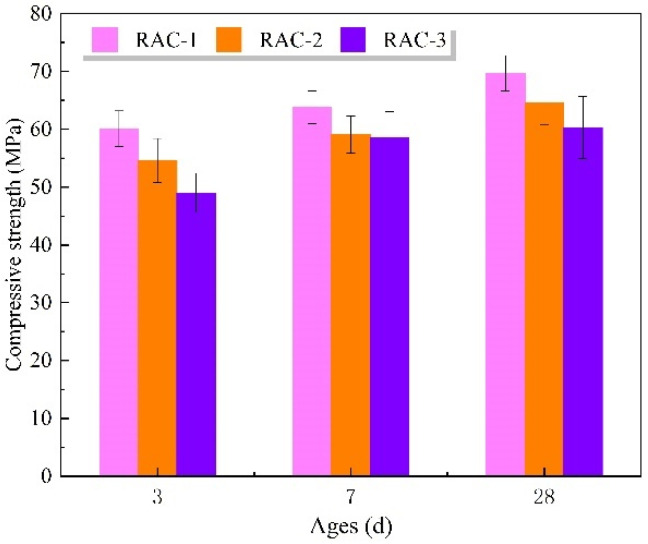
Compressive strength of concrete.

Assuming that the recycled aggregate absorbs water in fresh concrete, the actual w/c of RAC-1 is supposed to be lower than 0.24. Because of the lower w/c, the strength of RAC-1 is supposed to grow faster and should be higher than that of RAC-2 within 3–7 days. Then, because of the low water consumption, the cement of RAC-1 was not fully hydrated, resulting in a lower compressive strength than that of RAC-2 at 28 days [51]. This is contrary to the experimental results shown in Figure 10, where the actual test results show that the compressive strength of RAC-1 is consistently higher than that of RAC-2 within 28 days. This supports the conclusion of the LF-NMR imaging study that the *WAC* in fresh concrete is actually difficult to be fully absorbed by the recycled aggregate. It is worth noting that high strength recycled concrete has a low w/c and therefore the *WAC* has a greater effect on the w/c. The w/c of RAC-2 and RAC-3 is increased by 33% compared to that of RAC-1, which is the main reason for the decrease in compressive strength.

The compressive strength of RAC-3 was lower than that of RAC-2 with the addition of the same amount of *WAC*. There are two possible reasons: the first is that after 30 min of water absorption, the water film still exists on the surface of the recycled aggregate of RAC-3, which leads to the increase of water–cement ratio in the interfacial transition zones of recycled concrete and weakens the strength [50]; the second is that during the curing process, the relative humidity inside the concrete decreases as the hydration of cement proceeds, and the water absorbed by the recycled aggregate may seep out and weaken the ITZ [43].

## 4. Conclusions

The following conclusions were drawn from this study.

(a) The 1D frequency coding sequence of LF-NMR can be applied to characterize the water absorption behavior of recycled aggregates, and the CPMG sequences can characterize the effect of recycled aggregates on cement hydration. LF-NMR imaging is a viable method for continuous nondestructive measurements.
(b) When SRA is in contact with pure water, the depth of water migration in SRA increases with time and finally tends to be constant. When SRA is in contact with fresh mortar, little water migration occurs because the hydration of the cement in the mixture consumes a large amount of water, resulting in the inability of water to migrate through the capillary pressure into the recycled aggregate.
(c) For the paste with high w/c, the addition of SRA with different strengths and water contents has a significant effect on cement hydration. Meanwhile for the low w/c paste, the strength and water content of SRA are of little impact on cement hydration. Low-strength recycled aggregates in the saturated face-dry state have the greatest adverse effect on cement hydration, and dry, high-strength recycled aggregates have almost no effect on cement hydration.
(d) For high-strength RAC with a low w/c, *WAC* should be disregarded if the RCAs are strong enough and applied to fresh concrete in a dry state; otherwise, the compressive strength of the concrete will be reduced.

## Figures and Tables

**Table 1:** Chemical composition of white cement (%).

Composition	CaO	SiO_2_	MgO	SO_3_	Al_2_O_3_	K_2_O	Na_2_O	Fe_2_O_3_
P.W 42.5	68.352	21.169	1.639	4.709	2.778	0.382	0.471	0.223
P.W 52.5	72.172	15.956	3.909	3.835	1.844	0.804	0.274	0.267

**Table 2:** Physical properties of RCA-1 and RCA-2.

Coarse Aggregate	Grading (mm)	Apparent Density (kg/m^3^)	Crush Index (%)	Water Absorption (%)
RCA1	5~16.5	2580	10.6	6.4
RCA2	16.5~31.5	2590	10.6	4.7

**Table 3:** Mixture compositions for CPMG test.

Sample	w/c of Cement Paste	Water Content of SRA	Strength of SRA
Water-D-C25	Water replace paste	dry	C25
Water-D-C40	Water replace paste	dry	C40
WC03-D-C25	0.3	dry	C25
WC03-D-C40	0.3	dry	C40
WC03-S-C25	0.3	SSD	C25
WC03-S-C40	0.3	SSD	C40
WC05-D-C25	0.5	dry	C25
WC05-D-C40	0.5	dry	C40
WC05-S-C25	0.5	SSD	C25
WC05-S-C40	0.5	SSD	C40
WC03	0.3	\	\
WC05	0.5	\	\

**Table 4:** Mix proportions of concrete (kg/m^3^).

Sample	Cement	Silica fume	Sand	RCAs	Water	PS	w/b
RAC-1	520	57	709	990	138	2.60	0.24
RAC-2	520	57	709	990	183	1.56	0.32
RAC-3	520	57	709	990	183	1.56	0.32

**Table 5:** Statistics of the results of SRA moisture migration.

Sample	WC03	WC05	Water
C25	negative	negative	positive
C40	negative	negative	positive

## Data Availability

Not applicable.

## References

[1] Cakir O. (2014). Experimental analysis of properties of recycled coarse aggregate (RCA) concrete with mineral additives. Constr. Build. Mater..

[2] Piccinali A., Diotti A., Plizzari G., Sorlini S. (2022). Impact of Recycled Aggregate on the Mechanical and Environmental Properties of Concrete: A Review. Materials.

[3] Rakesh Kumar Reddy R., Yaragal S.C. (2023). A novel approach for optimizing the processing of recycled coarse aggregates. Constr. Build. Mater..

[4] Amario M., Rangel C.S., Pepe M., Toledo Filho R.D. (2017). Optimization of normal and high strength recycled aggregate concrete mixtures by using packing model. Cem. Concr. Compos..

[5] Du T., Chen J., Qu F., Li C., Zhao H., Xie B., Yuan M., Li W. (2022). Degradation prediction of recycled aggregate concrete under sulphate wetting–drying cycles using BP neural network. Structures.

[6] Lei B., Xiong Q., Zhao H., Dong W., Tam V.W.Y., Sun Z., Li W. (2023). Performance of asphalt mortar with recycled concrete powder under different filler-to-asphalt weight ratios. Case. Stud. Constr. Mat..

[7] Lei B., Yu H., Guo Y., Dong W., Liang R., Wang X., Lin X., Wang K., Li W. (2023). Fracture behaviours of sustainable multi-recycled aggregate concrete under combined compression-shear loading. J. Build. Eng..

[8] Lei B., Yu H., Guo Y., Zhao H., Wang K., Li W. (2023). Mechanical properties of multi-recycled aggregate concrete under combined compression-shear loading. Eng. Fail. Anal..

[9] Zhang H., Xu X., Liu W., Zhao B., Wang Q. (2022). Influence of the moisture states of aggregate recycled from waste concrete on the performance of the prepared recycled aggregate concrete (RAC)—A review. Constr. Build. Mater..

[10] Soares D., de Brito J., Ferreira J., Pacheco J. (2015). Use of coarse recycled aggregates from precast concrete rejects: Mechanical and durability performance (vol 71, pg 263, 2014). Constr. Build. Mater..

[11] Li J., Xiao J., Sun Z. (2004). Properties of Recycled Coarse Aggregate and Its Influence on Recycled Concrete. J. Build. Mat..

[12] Xiao J., Li W., Fan Y., Huang X. (2012). An overview of study on recycled aggregate concrete in China (1996–2011). Constr. Build. Mater..

[13] Maimouni H., Remond S., Huchet F., Richard P., Thiery V., Descantes Y. (2018). Quantitative assessment of the saturation degree of model fine recycled concrete aggregates immersed in a filler or cement paste. Constr. Build. Mater..

[14] Bentz D.P., Snyder K.A. (1999). Protected paste volume in concrete—Extension to internal curing using saturated lightweight fine aggregate. Cem. Concr. Res..

[15] Castro J., Keiser L., Golias M., Weiss J. (2011). Absorption and desorption properties of fine lightweight aggregate for application to internally cured concrete mixtures. Cem. Concr. Compos..

[16] Fan C.C., Huang R., Hwang H., Chao S.J. (2016). Properties of concrete incorporating fine recycled aggregates from crushed concrete wastes. Constr. Build. Mater..

[17] Wang Y., Cao Y., Zhang P., Ma Y., Zhao T., Wang H., Zhang Z. (2019). Water absorption and chloride diffusivity of concrete under the coupling effect of uniaxial compressive load and freeze-thaw cycles. Constr. Build. Mater..

[18] Yu Y., Wang J., Wang N., Wu C., Zhang X., Wang D., Ma Z. (2021). Combined Freeze-Thaw and Chloride Attack Resistance of Concrete Made with Recycled Brick-Concrete Aggregate. Materials.

[19] (2011). Pebble and Crushed Stone of Construction.

[20] (2013). Concrete-Specification, Performance, Production and Conformity.

[21] Leite M.B., do Figueire J.G.L., Lima P.R.L. (2013). Workability study of concretes made with recycled mortar aggregate. Mater. Struct..

[22] Mefteh H., Kebaili O., Oucief H., Berredjem L., Arabi N. (2013). Influence of moisture conditioning of recycled aggregates on the properties of fresh and hardened concrete. J. Clean. Prod..

[23] Agrela F., Sanchez de Juan M., Ayuso J., Geraldes V.L., Jimenez J.R. (2011). Limiting properties in the characterisation of mixed recycled aggregates for use in the manufacture of concrete. Constr. Build. Mater..

[24] Poon C.S., Shui Z.H., Lam L., Fok H., Kou S.C. (2004). Influence of moisture states of natural and recycled aggregates on the slump and compressive strength of concrete. Cem. Concr. Res..

[25] Tam V.W.Y., Gao X.F., Tam C.M., Chan C.H. (2008). New approach in measuring water absorption of recycled aggregates. Constr. Build. Mater..

[26] Li Z., Liu J.P., Xiao J.Z., Zhong P.H. (2019). A method to determine water absorption of recycled fine aggregate in paste for design and quality control of fresh mortar. Constr. Build. Mater..

[27] Feng Q., Liu X.-J., Peng Z.-G., Huo J.-H., Zheng Y., Liu H. (2021). Characterization of the influence of Nanoparticles on Early Hydration of Oil Cement by Using Low Field NMR. Energ. Source Part A.

[28] She A.M., Ma K., Liao G., Yao W., Zuo J.Q. (2021). Investigation of hydration and setting process in nanosilica-cement blended pastes: In situ characterization using low field nuclear magnetic resonance. Constr. Build. Mater..

[29] She A., Yao W. (2010). Probing the hydration of composite cement pastes containing fly ash and silica fume by proton NMR spin-lattice relaxation. Sci. China Technol. Sci..

[30] Gummerson R.J., Hall C., Hoff W.D., Hawkes R., Holland G.N., Moore W.S. (1979). Unsaturated Water-flow within Porous Materials Observed by NMR Imaging. Nature.

[31] Kopinga K., Pel L. (1994). One-dimensional Scanning of Moisture in Porous Materials with NMR. Rev. Sci. Instrum..

[32] Leech C., Lockington D., Dux P. (2003). Unsaturated diffusivity functions for concrete derived from NMR images. Mater. Struct..

[33] Brand A.S., Roesler J.R., Salas A. (2015). Initial moisture and mixing effects on higher quality recycled coarse aggregate concrete. Constr. Build. Mater..

[34] Therene F., Keita E., Nael-Redolfi J., Boustingorry P., Bonafous L., Roussel N. (2020). Water absorption of recycled aggregates: Measurements, influence of temperature and practical consequences. Cem. Concr. Res..

[35] Zhao H., Wu X., Huang Y., Zhang P., Tian Q., Liu J. (2021). Investigation of moisture transport in cement-based materials using low-field nuclear magnetic resonance imaging. Mag. Concrete. Res..

[36] van der Heijden G.H.A., Huinink H.P., Pel L., Kopinga K. (2011). One-dimensional scanning of moisture in heated porous building materials with NMR. J. Magn. Reson..

[37] (2016). Standard for Test Method of Performance on Ordinary Fresh Concrete.

[38] (2019). Standard for Test Methods of Concrete Physical and Mechanical Properties.

[39] Kim Y., Hanif A., Kazmi S.M.S., Munir M.J., Park C. (2018). Properties enhancement of recycled aggregate concrete through pretreatment of coarse aggregates—Comparative assessment of assorted techniques. J. Clean. Prod..

[40] Koenders E.A.B., Pepe M., Martinelli E. (2014). Compressive strength and hydration processes of concrete with recycled aggregates. Cem. Concr. Res..

[41] deOliveira M.B., Vazquez E. (1996). The influence of retained moisture in aggregates from recycling on the properties of new hardened concrete. Waste Manag..

[42] Domagala L. The Effect of Lightweight Aggregate Water Absorption on the Reduction of Water-Cement Ratio in Fresh Concrete. Proceedings of the 7th Scientific-Technical Conference Material Problems in Civil Engineering (MATBUD).

[43] Ferreira L., de Brito J., Barra M. (2011). Influence of the pre-saturation of recycled coarse concrete aggregates on concrete properties. Mag. Concrete. Res..

[44] Eguchi K., Teranishi K., Narikawa M. (2003). Study on Mechanism of Drying Shrinkage and Water Loss of Recycled Aggregate Concrete. J. Struct. Constr. Eng..

[45] Omary S., Ghorbel E., Wardeh G. (2016). Relationships between recycled concrete aggregates characteristics and recycled aggregates concretes properties. Constr. Build. Mater..

[46] Hu J., Ge Z., Wang K.J. (2014). Influence of cement fineness and water-to-cement ratio on mortar early-age heat of hydration and set times. Constr. Build. Mater..

[47] Zhou C., Ren F., Zeng Q., Xiao L., Wang W. (2018). Pore-size resolved water vapor adsorption kinetics of white cement mortars as viewed from proton NMR relaxation. Cem. Concr. Res..

[48] Jin D., Lang Z., Yao W. (2019). Analysis of Early Performance of Cement Paste by Low Field NMR. Appl. Sci..

[49] Lin Y.H., Tyan Y.Y., Chang T.P., Chang C.Y. (2004). An assessment of optimal mixture for concrete made with recycled concrete aggregates. Cem. Concr. Res..

[50] Al-Bayati H.K.A., Das P.K., Tighe S.L., Baaj H. (2016). Evaluation of of various treatment methods for enhancing the physical and morphological properties of coarse recycled concrete aggregate. Constr. Build. Mater..

[51] Xiao J., Tang Y., Chen H., Zhang H., Xia B. (2022). Effects of recycled aggregate combinations and recycled powder contents on fracture behavior of fully recycled aggregate concrete. J. Clean. Prod..

